# What drives the consistent use of long-lasting insecticidal nets over time? A multi-method qualitative study in mid-western Uganda

**DOI:** 10.1186/s12936-016-1101-4

**Published:** 2016-01-28

**Authors:** Clare E. Strachan, Anthony Nuwa, Denis Muhangi, Albert P. Okui, Michelle E. H. Helinski, James K. Tibenderana

**Affiliations:** Malaria Consortium Africa Office, Plot 25, Upper Naguru East Rd, P.O. Box 8045, Kampala, Uganda; Department of Social Work and Social Administration, Makerere University, P.O. Box 7062, Kampala, Uganda; National Malaria Control Programme, Ministry of Health, Plot 6, Lourdel Rd, P.O. Box 7272, Kampala, Uganda; European and Developing Countries Clinical Trials Partnership, P.O. Box 93015, 2509 AA The Hague, The Netherlands; Independent public health consultant, 74a Elderfield Road, London, E5 0LF UK

**Keywords:** LLINs, Malaria, Most significant change, Positive deviance, Qualitative, Social and behaviour change, Triangulation, Uganda

## Abstract

**Background:**

The distribution of long-lasting insecticidal nets (LLINs) through universal coverage campaigns is a widely adopted approach for the prevention of malaria at scale. While post-distribution surveys play a valuable role in determining cross-sectional levels of LLIN retention and use, as well as frequently cited reasons for non-use, few studies have explored the consistency of LLIN use over time, within the expected lifespan of the net, and the factors which may drive this.

**Methods:**

In this qualitative study, 74 in-depth interviews were conducted with (male) household heads and (female) caregivers of children in LLIN recipient households, as well as community health workers, in Buliisa, Hoima and Kiboga districts in Uganda, 25–29 months following a LLIN mass campaign distribution. A triangulation approach to data analysis was taken, incorporating thematic analysis, most significant change and positive deviance.

**Results:**

The factors found to be most influential in encouraging long-term LLIN use were positive experience of net use prior to the distribution, and appreciation or awareness of a range of benefits arising from their use, including protection from malaria as well as importantly, other health, lifestyle, social and economic benefits. Social support from within the community was also identified as an important factor in determining continued use of LLINs. Net use appeared to be more consistent amongst settled urban and rural communities, compared with fishing, pastoralist, refugee and immigrant communities.

**Conclusions:**

A multitude of interplaying factors encouraged consistent LLIN use in this setting. Whilst the protection of malaria remains a powerful motivator, social and behaviour change (SBC) strategies should also capitalize on the non-malaria benefits of net use that provide a long-term rationale for consistent use. Where supplies are available, SBC campaigns should promote replacement options, emphasizing ongoing net care and replacement as a household responsibility, thus reducing dependence on free distributions. The triangulation approach to qualitative data analysis enabled increased confidence in the validity of findings and an enhanced contextual understanding of the factors promoting consistent net use in mid-western Uganda. The approach should be considered when designing future studies to explore factors driving net retention and use trends.

**Electronic supplementary material:**

The online version of this article (doi:10.1186/s12936-016-1101-4) contains supplementary material, which is available to authorized users.

## Background

The ownership and use of insecticide-treated nets (ITNs) has been shown in multiple settings across sub-Saharan Africa to reduce clinical episodes of malaria and all-cause child mortality [1, 2]. The distribution of long-lasting insecticidal nets (LLINs) in particular through universal coverage campaigns (UCC) is a widely adopted approach and many countries, including Uganda, have seen considerable LLIN distribution activity in recent years. Post-distribution assessment has seen a growth in the utilization of qualitative methodologies over the last decade which has helped the exploration of social cultural beliefs and practices among poor, particularly rural, populations in high-risk malaria transmission areas, and their interplay with the use and non-use of nets. A range of factors seemingly influence LLIN retention and use such as climate and mosquito density, community norms and values, intra-family dynamics regarding decision taking and gender or age priorities, and those associated with the net itself such as age or physical condition [3–10]. Qualitative evaluations have however tended to remain sub-components of quantitative surveys [9] which are often conducted at specific time points post-distribution considered optimal for measuring net retention and use, such as within the first year following distribution and during the transmission season. While such surveys can play a valuable role in determining cross-sectional levels of LLIN retention and use, and frequently cited reasons for non-use [9], few studies have explored the consistency of LLIN use over the longer term, relating to the field-level expected lifespan of the net (2–4 years [11–13]), and importantly the factors which may affect this. Diffusion of innovation models illustrate the challenge in maintaining consistent use of any health ‘innovation’ even when use on adoption is high [14]. While some non-use of LLINs is, therefore, expected over time, such inquiry is valuable for informing ongoing effective social and behaviour change (SBC) strategies to promote consistent net use, as well as the design of appropriate follow-up LLIN supply strategies.

A qualitative study was conducted in Uganda to identify factors which may drive consistent or changing patterns of net use over time following a UCC. An approach involving multiple methods of qualitative analysis of in-depth interview (IDI) data was taken with the aim of gaining further insight into and contextual understanding of community perspectives, as well as optimizing the exploration of the data collected. Specifically, through triangulation of the results from multiple analytical approaches, the aim was to maximize data ‘confirmation’ (in this case, validation through repeated emphasis) and ‘completeness’ (detailed review of the full dataset) [15]. In addition to thematic analysis, the transcript data was evaluated using the most significant change (MSC) and positive deviance analysis approaches. MSC is a dialogical, story-based evaluation technique which uses inductive enquiry in search of ‘impact’, enabling further insight into aspects of positive or negative change and thus overarching lessons learnt [8]. The MSC technique requires a participatory analysis involving a systematic selection of the most significant of the stories collected by panels of designated stakeholders, enabling discussion of the value of reported changes among key groups, encouraging people to focus their attention on project impact, as felt by the beneficiaries, and to recognize that choices are based on individual preferences [8]. A key purpose of MSC is also to facilitate programme improvement by focusing the direction of work towards explicitly valued directions and away from less valued direction, the point being that both the content of the selected stories and the reasons for choosing them make the values of the different stakeholders explicit, and this can be used to foster dialogue between potentially competing perspectives [16]. The central premise of a positive deviance approach is that solutions to problems faced by a community often exist within that community, and that certain members possess wisdom, knowledge or positive attitudes which can be generalized to improve the performance or positive behaviour of other members [17, 18]. Support strategies that build on these ‘resources’ which already exist in the community have the potential to be more easily adopted and over a more sustained period [19].

This paper serves to highlight the value of the multi-method approach adopted to qualitative data analysis in the context of understanding the interplay of multiple factors which influence the use of LLINs over the lifespan of the net.

## Methods

### Study context

The study was conducted in the districts of Buliisa, Hoima and Kiboga (since separated into two districts, Kiboga and Kyankwanzi), in the mid-western region of Uganda. All districts had received LLINs through a UCC between November 2009 and March 2010, under the Malaria Consortium implemented Pioneer project and in collaboration with the Ministry of Health. Cross-sectional quantitative surveys indicated that while net ownership and use initially increased, it trailed off over time; household ownership of at least one net rose from 22.2 % prior to the campaign to 91.6 % 7 months post-distribution, then fell to 79.4 % 18 months post-distribution, and 62.2 % 36 months post distribution; and the proportion of children under five sleeping under an ITN the previous night rose from 13.7 % at baseline to 68, 59.6 and 45.2 % respectively [Malaria Consortium, unpublished data]. To enable exploration of the factors driving this trend, the qualitative data for this study was collected in April and May 2012, or 25–29 months post the UCC.

### Study design and sampling

Data was collected through IDIs, considered appropriate for understanding the perspectives of individuals and acquiring narratives of their experiences in using nets over time [20]. Sampling strata were identified from review of the quantitative survey data and from discussions with key informants from the district leadership. The aim of strata selection was to acquire an optimum range of experiences and perspectives with regard to net use over time, within the local population.

Specific sub-counties within each of the three districts were purposively selected for maximum variability in socio-economic, geographic, demographic and cultural context, considering; (i) urban/rural (based on distance from municipalities/trading centres which also generally corresponded to near/far proximity to health facilities and higher/lower socio-economic settings), (ii) major economic activities (fishing, pastoralists, farming/crop cultivation communities, traders), (iii) population composition (migrant groups, refugee settlements) (Table 1). Five sub-counties were selected per district, yet stratification within these was not uniform given the considerable contextual variation. One village was selected per sub-county.

**Table 1:** Location of study sites and strata samples; the number of interviews conducted is indicated

District	Sub-county	Village	Strata	No. interviews conducted
Hoima	Central Ward	Kijungu A	Urban	4
	Kitooba	Mpunda	Rural pastoralist	5
	Kyangwali	Kasonga	Refugees settlement	5
	Buseruka	Toonya	Fishing community	5
	Kiziranfumbi	Kidoma	Immigrant population	5
Buliisa	Buliisa	Bugana	Rural pastoralist	5
	Biiso	Biiso trading centre	Urban	5
	Butiaba	Piida A	Fishing community	5
	Kihungya	Nyeramya	Community in lower escarpment	5
	Ngwendo	Ngwendo Farm	Rural crop cultivators	5
Kiboga/Kyankwanzi	Bukomero	Bukomero A	Urban	5
	Ntwetwe	Natolye	Part urban/rural	5
	Kyankwanzi	Kayanja	Rural pastoralist	5
	Lwamata	Nsanje	Rural cultivators	5
	Butemba	Byerima	Rural (hard to reach)	5
Total				74

Within each village, five households were sampled for IDIs (one per household); informants included two male heads of households, two female caregivers of children, and one community health worker [known as village health team (VHT) members in Uganda], with the aim of capturing a range of community perspectives. In one village, there was no VHT available for interview and so just four households were sampled. For the male and female informants, households were sampled at random so as to avoid a potentially biased selection of informants. As household lists were not available, the ‘random walk’ [21] approach was used, whereby a bottle was spun from the middle of the village which indicated the direction to walk and specific households were selected based on randomly chosen ‘intervals’ (i.e. 5, 24) depending on the size of the village and as determined by counting households to the boundary. VHTs were selected at random from a list of active VHTs available from the village leadership. Households were only included if they had benefitted from the UCC of LLINs (without observing that the net was present). If saturation of emerging themes was not considered to have been reached based on a review of transcripts of the existing sample, further households would have been selected though this was not perceived as necessary.

### Data collection

A semi-structured topic guide was used to explore factors which could contribute to use or non-use of nets, as well as changes in net use behaviour over time, the value placed on net use, and expectation for continued net use in the future. Additional questions on the most significant change(s) (i.e. MSC methodology) resulting from the receipt and use of LLINs were added to the topic guide. The same topic guide was used across each target group. The tool was pre-tested in a comparable context and adjusted as necessary before initiation of data collection. All interviews were audio recorded, with notes written for back up. Verbatim transcripts were drafted in the field as soon as possible after each interview. All transcripts were translated from the local language into English during the transcription process. Back-translations were carried out on samples for quality control purposes.

### Data analysis

Three different approaches to the analysis of interview data were adopted, allowing for the triangulation of findings thus aiding the validity of the results generated. Firstly, a thematic analysis (which included data from the MSC enquiry) was conducted following a modified ‘Framework Approach’ [22], which started with a pre-existing coding frame based on the research questions, to which codes were added on review of the data. All data was coded and indexed in Excel and analysed according to the most salient themes.

Secondly, specific stories about the most significant change(s) informants had experienced from receiving and using a net were developed from each informant, utilizing the verbatim transcription of responses to the MSC enquiry whilst adding contextual information to enable a stand-alone ‘story.’ An iterative, stepped approach was used for the selection of MSC stories, with different stages of the selection conducted by different groups involved with the project (Table 2). During the selection process, each story was read out, and in-depth discussions took place relating to batches of five stories about the value of the reported changes with a view to exploring and learning about the project impact. The use of ‘domains’ (selection categories) can help guide the selection of MSC stories where there are a range of possible change ‘areas’ [23], but in this case, no domains were prior agreed so as to avoid narrowing the idea of ‘change’ and given all stories related to the same theme—‘use of nets for the reduction of malaria’. Stories from household informants (encompassing male household heads and female caregivers) were considered separately from those from VHTs given the former tended to focus on personal and household experiences and the latter on more community-wide changes. An ‘iterative voting’ approach (discussion after each vote in order to move towards consensus) [23] was used to agree on the stories to be taken to the next analysis stage, with the aim at the final analysis stage being to select one overall MSC story. Notes explaining the reasons for selection were taken for each included story. The MSC stories were further analysed using a summative approach, involving counting the number of times a specific change (relating to benefit or impact of net use) was mentioned across all stories.

**Table 2:** Results of stepped MSC story selection process

Selection/analysis phase	Team composition (no. of people)	No. of stories reviewed	No. of stories selected	Source of stories selected
1	Pioneer project team (4)	74	26	21 household informants, 5 VHTs
2	Malaria Consortium Uganda management team (4)	26	16	11 household informants, 5 VHTs
3	District stakeholders (District health team members and study participant/beneficiary representation) (4/2)	16	3	2 household informants, 1 VHT

Thirdly, ‘positive deviant’ behaviours from the full transcript data were identified and discussed among the research team. Data analysis under the positive deviance approach is usually conducted in accordance with standard principles for qualitative and/or quantitative analysis with a focus on the outcome of what constitutes performance [24]. In this study, the analysis attempted a reflection of what positive deviance may constitute considering ‘performance’ in terms of ‘consistent net use’.

### Ethics, consent and permissions

Ethical approval was acquired from the Uganda National Council for Science and Technology in Kampala, Uganda (no. HS 1009). The consent form contained information on the objectives of the survey, confidentiality, respondent rights and possible uses of the data. Interviews proceeded when verbal consent was provided.

## Results

A total of 74 IDIs were conducted, 30 with male heads of household and female caregivers respectively, and 14 with VHTs (Table 1).

## Thematic analysis

### Net usage

Net use behaviour reportedly varied amongst respondents, and ranged from consistent use to seasonal use to, in two cases, non-use. More than half of all households reported that all family members slept under nets most of the time. VHTs across strata suggested that community members regularly used their nets even when sleeping outside their homes, such as children at boarding schools or fishermen sleeping close to lake shores. In households where not all members slept under nets, this was mostly due to an insufficient number of nets, a growth in family size, households having not received enough nets during the UCC, or nets having since been destroyed or lost. Common household responses to an inadequate number of nets were the further sharing of sleeping spaces, or the prioritizing of family members, usually children or pregnant women.

Seasonal use of LLINs was reported across strata, and appeared to relate to the fluctuating visibility of mosquitoes and, therefore, perceived malaria threat, a decline or rise in local malaria cases, and seasonal temperature change which affected levels of comfort experienced when sleeping under the net.

"We were given these nets during the rainy season when there were a lot of mosquitoes. I hung the nets and the children slept in them that time. When the rainy season was over, we removed the nets because during that time there were no mosquitoes." (Male, Bugana village, Buliisa).

Net use appeared to be more consistent amongst settled urban and rural communities compared with fishing, pastoralist, and refugee/immigrant communities, where a number of respondents raised problems in hanging nets in round houses and a lack of proper sleeping beds, which in some cases reportedly led to nets tearing. In Piida A (a fishing village) and Kasonga (a refugee settlement), all household members used a net in just two of the five households, compared with Mpunda (rural poor community) where all members used nets in four out of five households, and Kijungu A (an urban wealthier site) where all members reportedly used nets in all households. The data suggested limited movement of nets across households (‘intra-household exchange’), with only two households having given away nets to other people.

Although reports were not widespread, there were some cases of nets being used for other purposes such as for fishing, drying silver fish, fencing for domestic animals, construction of bath shelters, fencing or shades for plants, and for make-shift curtains. Such cases were more common in fishing, pastoralist and immigrant communities. There was some but limited suggestion of nets from the UCC being sold to make money, though it was not clear whether these nets were in fact excess nets, and individuals who used nets for other purposes in fact also used them for malaria protection at home.

"There are people on landing sites who use nets for fishing. Some people sell nets in order to get some money while other people use nets to guard their crops from animals and other pests. People say that since these nets are treated, they are capable of chasing away animals." (Male, Nsanje village, Kiboga).

Overall, experience in using nets before the UCC appeared to contribute to people’s tendency to use nets in the long-term, seemingly because they have had more time to appreciate the benefits of net use, or simply because net use was already a more entrenched practice.

"Everyone in this family has a net and they all sleep under these nets because even before the government nets came, we had nets for everyone in the family so there has been no change." (Female, Kayanja village, Kiboga).

"I grew up seeing my parents and all of us in our home sleeping under the nets so it was something I had to emulate and practice when I got my own family" (Female, Toonya Village, Hoima).

### Perceived benefits of net use

Across strata, perceived benefits of net use appeared key in encouraging the consistent use of nets over time and, as expected, principally related to the prevention of mosquito bites and malaria. Similarly, previous bad experiences with malaria appeared to reinforce the incentive to continue using nets. Some households mentioned a seemingly comparative ineffectiveness of other malaria preventive methods used prior to receiving nets such as sprays, coils or slashing the compound, which appeared to have boosted their focus on and use of nets.

"When we slept in those nets that (first) night, me, my husband and the children …we slept comfortably because the mosquitoes didn’t get close to us - they had no chance to bite us. Even now, as we continue to use these nets, the situation is still the same as it was when we slept in these nets for the first time." (Female, Ngwendo Farm village, Buliisa).

"Before the introduction of nets, we were using other methods of fighting malaria which included lighting a candle at night to chase away mosquitoes, slashing around the compound and clearing any stagnant water near the house. Despite the efforts, all these methods failed to reduce malaria and that is when we decided to use nets… Malaria has now reduced due to the use of nets." (Female, Kidoma village, Hoima).

Beyond protection from malaria, additional benefits of net use most commonly mentioned, and which appeared to drive consistent use, included providing a barrier against other insects such as cockroaches, protection against falling grass and other debris from the roof, the warmth provided by nets (in some cases reducing the need for a blanket), less sleep disturbance and overall better skin and general health. The reduction in malaria was also reported to have afforded household members more time to work in their gardens or perform other income-generating or household duties. A few respondents also noted the aesthetic benefit of a net hung above the bed.

"The mosquitoes and insects which used to bite us or fall on us while sleeping, these days I don’t see them … so we sleep comfortably. We have peace now because the skins of my children are healthier and no longer have scars of mosquito bites. …sleeping under nets has increased my time to go to my gardens and do my domestic work without being tired and stressed." (Female, Kidoma village, Hoima).

### Social support and influence

Household heads and/or caregivers tend to allocate sleeping spaces and nets and, in that sense, were found to be in a strong position to influence net use. Their level of enthusiasm in encouraging members of the household, particularly children, to sleep under a net was found to be important in either boosting or reducing net use over time.

"Some children sleep carelessly, others sleep and kick these nets while sleeping and so as a parent I am forced to frequently wake up at night to check whether they are still sleeping in the nets and if not I have to cover them properly." (Female, Nyeramya village, Buliisa).

While personal or household experience in using nets seems to have been a key driver in promoting consistent net use, respondents were asked about any people or groups in their community who had appeared to encourage or discourage the use of nets. Encouragement for net use reportedly came mainly from village leaders (“local council [LC] 1 s”) and VHTs, who promoted net use at village meetings and conducted home visits where they would offer direct support in hanging and using the net. Government officials, religious leaders, health workers, neighbours, as well as non-governmental organization project staff were also mentioned in encouraging net use. Radio stations were also reported to have programmes and advertisements promoting net use. A small number (three) incidences were mentioned where people had seemingly discouraged net use through rumours or the sharing of bad experiences, largely relating to health concerns from the insecticide in the nets.

"A few days after were had hung the nets we got a rumour that these nets had medicine which could make us sick. This prompted us to remove the nets immediately. We put them back to their polythene packages and kept them in a safe place…It was [a] rumour circulating in the village but I cannot know who was saying it. At a later stage, I realized it was a lie and my children resumed using the nets." (Female, Bukomero A village, Kiboga).

### Net condition

As expected, people were more likely to use a net if it remained in good condition and less likely if it had developed holes or was torn and thus was deemed less effective in protecting them from malaria. Some respondents across strata mentioned that net condition had deteriorated over time as a result of poor bedding materials, such as papyrus reed mats, or rats. Few respondents (three) mentioned actual repair efforts though VHTs, more than household heads and caregivers, indicated a willingness to repair their nets to enable continued use. Among those who made attempts to repair the nets, it appeared that usage was only stopped when the net was considered beyond repair.

"I no longer use my net because it got holes and many mosquitoes started entering in it and I decided to remove it completely from my bed. …It is my grandchild who burnt many holes into my net. He was playing with a matchbox…it was hard for me to repair. That is when I decided to remove it." (Female, Bugana village, Buliisa).

### Availability and purchase of replacement nets

Across the different strata, and thus across a range in terms of access to services and potential ability to pay, once nets were considered old or damaged and thus less effective, people tended to stop using them or instead switched to untreated nets rather than source new LLINs. There were no examples of households having replaced their nets through the purchase of new LLINs using their own resources. This is despite some informants having bought nets before the UCC, although the proportion of those having been LLINs is likely to have been low. The few households which reported having acquired replacement nets on their own had bought untreated nets. When asked what they would do when their nets grew old, the most common response across strata was to wait for another free net rather than replace the net themselves; as well as the expectation of further net donations, an overall lack of funds and low availability through the local commercial sector appeared to contribute to this response.

"When the nets I have are worn out or even get burnt, I would inform the village leader who will inform the administrators to get us new nets. If we don’t get nets from the donors it would be difficult for me to buy a net because I don’t have money to buy a net." (Male, Kasonga village, Hoima).

"When the mosquito nets grow old, I will not buy nets because in the community there are no shops which sell nets. That is why am requesting the government to give us more nets. Secondly, so many organizations have been giving us free nets so if I buy any net I would be wasting money - I know they will keep coming to give us nets." (Female, Piida A Village, Buliisa).

### Other factors influencing net use

Other practical factors that appeared to influence net use, though perhaps more in the initial phase post-distribution than over the long-term, included an inadequate space to hang the net (i.e. small houses or bedrooms), a lack of proper beds (difficulties in spreading and tucking in nets when sleeping on the floor), and challenges in hanging rectangular nets in circular houses. Some households had also switched from LLINs to untreated nets due to perceived dangers or irritations with the insecticide in the LLINs, or because the netting material was perceived to be too coarse.

### Changes in net use experiences over time

Asked specifically about their household’s recent experiences in using nets compared with when they first received them, most respondents, across strata, relayed positive experiences, with few changes in net use experience over time. Many did mention though that nets became less potent and thus less effective over time causing the amount of visible mosquitoes in the house to rise again.

"Nothing has changed. I still use [nets] in the same way like I did in the past. What I can say is that now the nets are no longer that effective and mosquitoes enter and bite my children at night." (Female, Kijungu village, Hoima).

### Most significant change story selection and summative analysis

The story selection process followed three distinct stages with different groups of stakeholders (Table 2). The emphasis of the selection process was to establish what was considered significant in the changes reported and why, and thus selection was based on criteria generated inductively. These included: (i) stories that went beyond just malaria prevention and included wider social and economic benefits at the household level, (ii) stories that illustrated emotional and personal feelings (impacts) about the changes being reported; (iii) stories that explained the changed situation before and after use of LLINs, and (iv) stories that included reference to severe malaria. Of interest was that stakeholders at each selection stage adopted similar reasoning for the selection of stories. Stakeholders at all selection stages were particularly responsive to emotive stories in terms of suggesting them as ‘significant’.

**Fig. 1:**
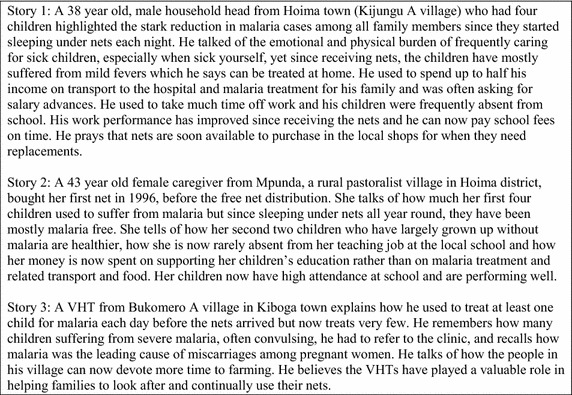
Summaries of most significant change (MSC) stories selected

The ‘significant changes’ reported to have resulted from the use of nets were wide-ranging and spanned direct health benefits, as well as economic, social and emotional benefits, felt at both individual and household levels. The three MSC stories finally selected (the final group were unable to agree on one MSC story from the three remaining) all reflect this range of benefits (Fig. 1), available in full (Additional file 1). The VHTs, in addition to the benefits to them as individuals and their families, spoke of community-wide and health system changes, such as reduced stock outs of anti-malarial drugs and shorter patient queues at health centres.

**Fig. 2:**
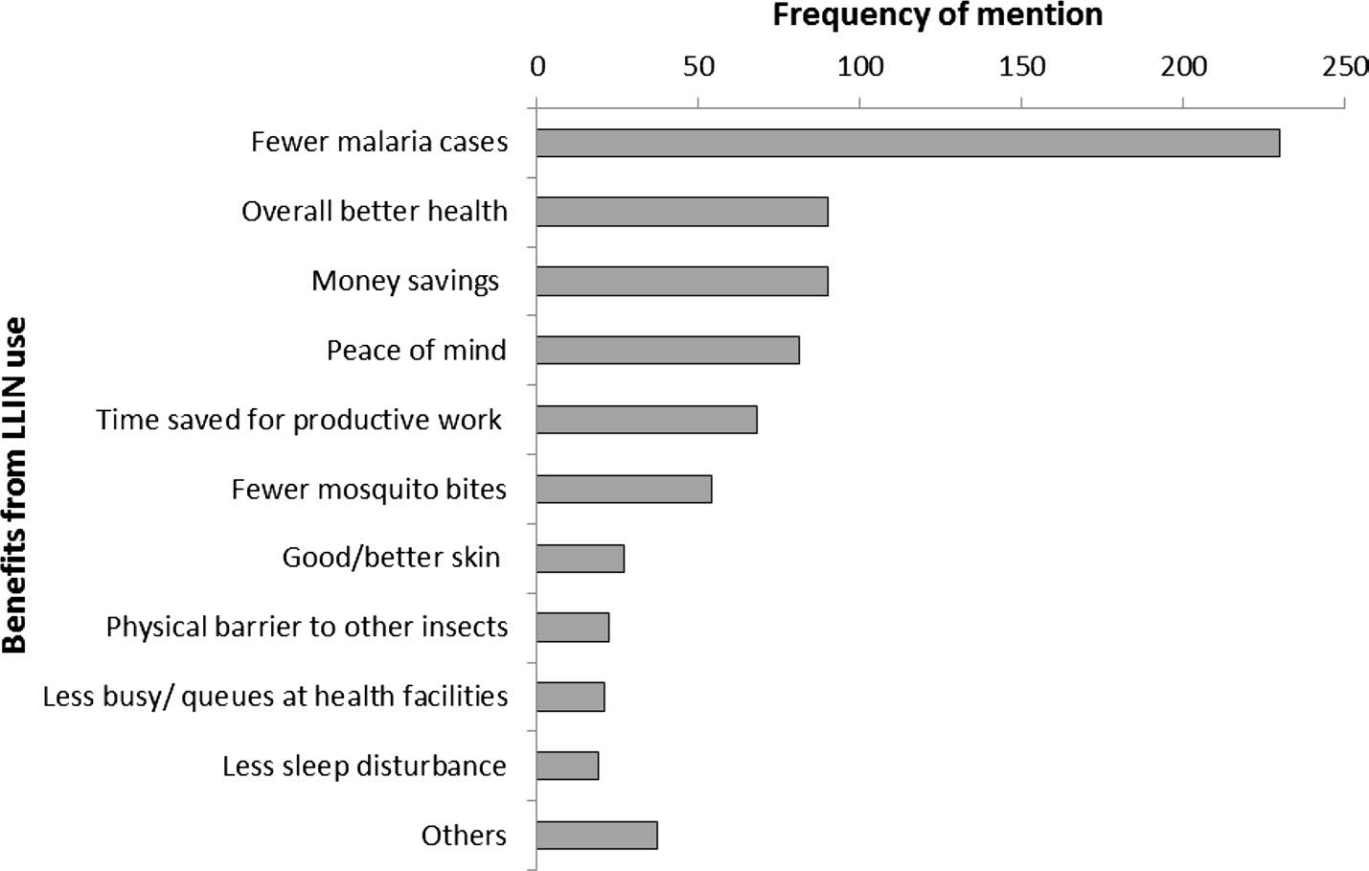
Frequency of ‘change’ mentions across all MSC stories

A summative analysis approach was also taken to review the frequency of most significant changes experienced from using nets since the UCC as mentioned across all MSC stories (Fig. 2). Overall, ‘fewer malaria cases’ was the most commonly mentioned change reported, corresponding with findings from the thematic analysis which suggested prevention of malaria as a key driver for consistent use. This was followed by ‘better overall health’ and ‘money savings from less need to access care/treatment for malaria’. ‘Peace of mind/less stress’ was the next most commonly mentioned change, an aspect also highlighted through the story selection process. These changes, and others, were all mentioned more frequently than ‘reduced severity of malaria’, though this would have been covered in part through ‘fewer malaria cases’. ‘Acting as a physical barrier from other insects or substances’ (i.e. debris) was mentioned less frequently than may have been anticipated from the results of the thematic analysis. In general, there appeared to be little difference in terms of the most commonly mentioned changes reported across strata, which corresponds to little variance in factors driving consistent net use across strata as reported from the thematic analysis.

### Positive deviance analysis

Patterns of net use behaviour varied widely from cases where all household members consistently used nets to cases where whole families had abandoned net use (these could be seen as negative deviants). Discussion gave focus to past (pre-distribution), current, and intended (future) behaviours as relating to consistent net use (Table 3). Characteristics of positive deviant respondent households (keen users) included having previously used nets before the UCC, use of nets throughout seasons, the adapting of sleeping spaces to enable covering of all family members, response to challenges to enable regular net use, motivation of others to use and care for their nets and they generally described nets as being an integral part of their lives. They also intended to continue using nets and would be willing to purchase a replacement. Many of these behaviours appeared to be mutually reinforcing i.e. family members share sleeping spaces so all members can sleep under a net each night and because net use has become habitual, it follows naturally to encourage or support others in using nets.

**Table 3:** Common characteristics of positive deviants

Past net use behaviour	Had nets (LLINs/non LLINs) before UCC (most likely purchased)
Current net use behaviour	Use nets all the time (nightly, all seasons) All household members sleep under a net Family members share sleeping spaces to enable all to be covered Have devised solutions to address practical challenges in net use i.e. nails/poles to hang nets properly and repairs Net use has become a habit/daily routine Carry a net when sleep elsewhere Encourage visitors to sleep under nets Support their neighbours to use and care for nets
Intended/future net use behaviour	Have intention to continue to use Willing to buy replacement nets

## Discussion

Data from three qualitative analytical approaches have collectively indicated that a multitude of factors appeared to drive the consistent use of LLINs over time in this setting. These relate to the perceived benefits of net use, previous experience with net use, the influence of household heads and caregivers as well as wider social support and influence, net preferences, net condition, availability of nets within the household, and cost and availability of replacement nets.

While there appeared to be little difference in terms of factors driving long-term net use across strata, so between varied geographic, socio-economic, demographic and cultural contexts, net use appeared to be more consistent among more ‘settled’ communities. Future net distribution and usage promotion programmes should effectively consider the practicalities of hanging and using nets across a range of household structures with varied sleeping arrangements.

Two factors emerged as key when exploring long-term LLIN use. Firstly, previous positive experience of net use, or positive experience in net use over time, seemed to lead to net use as part of a daily or habitual routine and implies an enhanced value placed on the net derived from continual reinforcement of benefit over time. The findings show that people who had bought and used nets before the UCC were more inclined to continue using nets. On the other hand, people with no history or experience of net use were more likely to use nets inconsistently or to abandon them altogether.

Secondly, perceived benefits of net use, which could translate into actual benefits, seemed to drive consistent net use. Perceived benefits largely related to protection from malaria, reinforced through previous negative household experience of suffering from malaria, but other health, lifestyle, social and economic benefits were also valued. The thematic analysis emphazised better health overall, avoidance of mosquito bites, less sleep disturbance, the creation of a barrier against other insects, as well as more time for income-generating activities. The summative analysis of the MSC stories gave emphasis to money saving, the opportunity to spend more time on productive work, as well as enhanced peace of mind from less sickness from malaria. This was further illustrated by the MSC selection process, which highlighted a range of personal and emotional responses to the impact of using nets over time. Other studies have highlighted the non-malaria benefits in using nets as perceived by communities; a study in The Gambia showed that privacy could be a motivating factor [25], and in Zanzibar, aspects of comfort (getting a good night’s sleep and avoiding biting pests) appeared to play a key role in personal decisions to use nets consistently [26]. While communication campaigns promoting net use have tended to focus on direct benefits in terms of malaria prevention, perhaps assuming this to be sufficient as a motivator, this study adds further evidence to suggest that future campaigns should capitalize on the non-malaria benefits of net use that provide a long-term rationale for consistent use.

The importance of emphasizing the non-health advantages to sleeping under nets have also been suggested in response to seasonal fluctuations in mosquito numbers which can affect the perceived threat of malaria and, by association, net use [27]. Seasonality emerged as a key factor preventing the consistent use of LLINs, and is a well-documented factor causing peaks and troughs in net use within endemic settings which experience seasonal fluctuations in malaria transmission and where the causal link between mosquito bites and malaria incidence is largely accepted [9, 28]. The observation that some VHTs, who are trained in and responsible for leading health promotion efforts in communities, also appear to use LLINs less in the dry season represents the scale of the challenge when encouraging net use throughout the year in such settings.

All analysis approaches emphasized the important role played by household heads and caregivers in encouraging and sustaining net use, as well as in the maintenance and care of nets. SBC efforts to promote ongoing community net use should (continue to) target both these groups. In addition, social support from within the community was identified as an important factor in determining continued use of LLINs, with LC1s and VHTs playing key roles in promoting habitual net use.

While most respondents reported a continuation of positive experiences from using LLINs over time, some also emphasized that the nets had appeared to become less potent, and thus less effective, over time. This could have been expected to cause a reduction in use, though surprisingly, there were no reports of related discontinued use, perhaps due to some appreciation of wider benefits as already discussed. However, discontinued net use did appear to result from deteriorated net condition, specifically from burning or holes which had otherwise developed. Children, rats and the type of bedding material used were identified as common causes of net damage, which are also implicated in other studies [29, 30]. Few attempts to repair the nets were reported although this study did not specifically set out to determine net repair behaviour. Work in another region in Uganda showed that exposure to SBC messages promoting net care and repair resulted in improved knowledge and attitudes towards care and repair, which impacted positively on net condition [31].

Continued LLIN use is closely related to ability to replace worn out nets. In this study, there were no reported cases of purchased replacement LLINs, even among those who valued their nets and discussed habitual net use, though there were two cases of purchase of untreated nets. The data suggested a significant expectation of, or hope for, another free distribution (a further UCC took place in November, 2013), though a lack of funds with which to purchase replacement nets and a lack of availability of LLINs (or even untreated nets) in the commercial sector was also apparent. Policy decisions are required to support the availability of replacement LLINs through both public and private channels. If a ready supply is available, SBC communication campaigns should promote replacement options, emphasizing ongoing net care as well as replacement as a household responsibility and thus reducing dependence on free distributions.

The triangulation of the results from multiple analytical approaches, including thematic analysis, MSC evaluation and the identification of positive deviance behaviour, helped build an enhanced contextual understanding of the factors which appear to promote consistent net use in this setting, and to validate findings across the dataset. The MSC technique effectively documented the community’s understanding of what constituted significant change as arising from owning and using LLINs. The stepped analysis approach enabled a range of key stakeholders in project, administrative and managerial positions and project beneficiaries themselves to reflect on the range of benefits felt as a result of ongoing net use. The MSC story selection process helped inform ongoing implementation under the Pioneer project, specifically through incorporation of a wider range of benefits of net use during SBC community dialogue discussions, and also helped set the parameters and scope of enquiry for the endline project assessment. Given the limited published work documenting the use of MSC in public health impact assessment [32, 33], is it hoped that this study will encourage its wider use, particularly in studies adopting a triangulation approach to the analysis of qualitative data.

Positive deviance is a behaviour change approach that builds on strengths that exist in communities, and has the potential to accelerate the adoption of desired behaviours [17, 18]. Positive deviance has been successfully applied in public health programmes for nutrition, and maternal and newborn health, among others [34]. Its use in malaria prevention and control is limited, though encouraging results were obtained in a study in Cambodia that used role model migrant workers as change agents for better prevention and health-seeking behaviours in the community [35]. Positive deviance analysis of net use in this study explored behaviours at the point when households place high value on their net and when regular net use is an adopted habit. An understanding of the drivers of positive deviant behaviour, and further analysis of how these individuals have overcome challenges and found solutions, can be valuable in the design of effective SBC programmes and other LLIN distribution supportive strategies. Positive deviants also have the potential to be powerful advocates within their community.

## Limitations

The study had a number of limitations. Many respondents had difficulty in reflecting on behaviours over a period of time, focusing more on the immediate aftermath of the UCC and the present; the use of event calendars may assist with recall in future studies of this nature. Capturing information relating to behaviours where there are potential incentives at play (i.e. for household informants, demonstration of gratitude for a freely provided net or to convey the need for a replacement net, or for VHTs, illustration of their example-setting behaviour within the community) proved to be challenging, despite having processes in place to ensure confidentiality; some social desirability bias must therefore be considered. The number of respondents to reveal consistent non-use of their LLINs was small. A detailed exploration of factors driving non-use over time would also be valuable for the design of locally-appropriate SBC strategies, as well as effective distribution approaches; future studies could consider oversampling from the non-use group. Similarly, while the positive deviance analysis provided a valuable insight into the characteristics of consistent net users, positive (or negative) deviance analyses are strengthened from the use of large samples which enable a detailed assessment of what can be minority group(s). Finally, the interview tool was not translated into the local languages because they are more commonly spoken than written, and thus emphasis was placed on ensuring field researcher comprehension of the meaning of questions to enable effective on-the-spot translation. Though this approach was considered preferable in this setting, it is possible that this could have resulted in the loss of some nuanced expression.

## Conclusions

The factors which emerged as key in driving long-term LLIN use included previous positive experience of net use, or positive experience in net use over time, and perceived benefits of net use, which could translate into actual benefits, including protection from malaria as well as other health, lifestyle, social and economic benefits. The results highlight a number of important learnings for consideration when designing SBC strategies in mid-western Uganda and other comparable settings, notably promotion of the consistent use of nets throughout the year, the importance of including household heads and caregivers as key agents in sustaining net use behaviour, and the promotion of viable, affordable options for the replacement of nets where the supply is available. Emphasis should also be given to the wider benefits of using nets, as experienced by targeted communities, beyond the prevention of malaria. ‘Positive deviants’ have the potential to play a valuable role in the promotion of ongoing net use in communities and further analysis of a larger positive deviance sample could enable more detailed insight into anticipated challenges and solutions relating to consistent net use, valuable for the design of a range of supportive strategies. The multi-method approach to qualitative analysis, incorporating thematic analysis, MSC and positive deviance, enabled an enhanced contextual understanding of the factors which promote consistent net use in this setting, as well as increased confidence in the validity of the findings through data triangulation. The approach has merit and should be considered when designing future studies to explore factors driving net retention and use trends.

## Additional file

10.1186/s12936-016-1101-4 Full transcripts of the three stories selected as part of the study’s MSC analysis component.

## Abbreviations

IDI: in-depth interview
ITN: insecticide-treated nets
LC: local council
LLIN: long-lasting insecticidal nets
MSC: most significant change
SBC: social and behaviour change
UCC: universal coverage campaign
VHT: village health team
