# Supplementary material for: What drives the consistent use of long-lasting insecticidal nets over time? A multi-method qualitative study in mid-western Uganda
Source: Malar J. 2016 Jan 28;15:44. doi: 10.1186/s12936-016-1101-4 (PMC4730749; doi:10.1186/s12936-016-1101-4)
Supplement: Supplementary file 1 — 10.1186/s12936-016-1101-4 Full transcripts of the three stories selected as part of the study’s MSC analysis component. [file 12936_2016_1101_MOESM1_ESM.docx]

**Additional file 1**

Box 1: Male household head, Kijungu village, Hoima (story no. 38).

*“I have four children. The eldest is 18 years and the youngest is five years. The nets have helped us reduce the number of times we suffer from malaria. When I look back when I was not using the nets, I thank whoever introduced those nets. Mosquitoes are still many but at least we sleep in nets [and] they cannot bite us when we are sleeping unless when we have not gone to bed, that is when they get the chance to bite us. As you can see the place we are staying in though it is [within] the town we are surrounded by a bush on undeveloped plots. There are many mosquitoes because of the over grown bushes that have created a good environment for mosquitoes to breed in. Before we started using nets we were frequent visitors at the main hospital. Every time we would be sick from malaria and would be admitted for malaria. Malaria used to disturb the children a lot compared to the adults. They would reach to an extent of collapsing – convulsing - with high fevers. I remember one time when we were admitted and discharged. When we came back home that very day we found another child having a high temperature and was convulsing we had to rush back to the hospital for admission. Really it was a tiring situation for me and my wife.*

*When they were admitted I had to request at my work place to be allowed to take care of them. While my wife was attending to the sick in the hospital I had to seek absence from office in order to look after those children who were at home. I had to shuttle between home and the hospital to take what they needed to use and taking them food. Sometimes I had to send my wife back home for her to have a rest lest she collapsed because of taking care of the sick. At times two children would be admitted at the same time and other times others would not be admitted but they would remain home sick. Every time I would be asking for salary advance to meet treatment costs and other costs like transporting the sick to the hospital. This would affect my salary because I had to spend more than half of it treating my people at home. At my work they had started doubting my performance because I was always having sick children and asking permission to take care of them. My children would miss school for two or three weeks in a term because of malaria. I would say the situation was only in my home but even in other people’s homes in the neighbourhood, [they] had the same problem. We could meet with my neighbours with sick children from malaria at the hospital.*

*We no longer have the above scenario and my children are happy. When we started using the nets in 2010 my youngest daughter was three years old. Since then she has never fallen sick of malaria. My other children since then have also not got malaria. Even we the adults have not had malaria. The children these days suffer from minor fever which is brought by flu and cough. This one is treated at home and it does not stop the children from attending school or stopping me from going to work. My performance at work has improved. I no longer ask salary advance as I used to do in the past. I can pay my children’s school fees in time compared to when my children could be chased from home all the time. If we continue using the nets we will not suffer from malaria in the future. We have continued sleeping in these nets and we shall continue using them in all weather because malaria has no season. I think these nets should be made accessible cheaply in shops which are everywhere and near us so that we can afford to buy them in the future. I only pray the situation continues like this.”*

Box 2: Female caregiver, Mpunda village, Hoima (story no. 43).

*“I have stayed in this village for 43 years. I was born in this village and married in the same village. I was married to my husband 22 years ago. By then I was teaching as a licensed teacher in a primary school near our home. I continued teaching in the same school until I retired early. In this village malaria was a lot among the adults and children. Children used to die a lot from malaria, mostly those below six years. When I got married I knew too my children will suffer from malaria. Indeed my first four children too got their fair share of malaria when they were born.*

*Changes to me go back to 1996. So when you mention changes in two years I wonder if you are aware I have used the nets longer than that. The first change was malaria reduced in the home. My children and us - father and mother - no longer suffer from malaria. We kicked it out of our home. Secondly the money which we used to spend on treating ourselves has gone into educating our children. Thirdly when I started using the nets, my performance at school where I was teaching improved. I was no longer absent from duty and my school was happy. My children would attend their school term without staying home because of malaria. Because we started using nets at home, by the time I had my last two children they never suffered from malaria. Up to now they are very different from the first four children. They have been healthy through their lives because malaria has not affected them at all. Lastly, money which we used to spend on treatment and transport every time when anyone got sick is now used to pay fees in time for our children.*

*The four children got malaria on and off. However, in those four there was one particular child who disturbed us a lot. Whenever she got malaria, the fever would go high and she would convulse. I was in hospitals for admission on and off. Sometimes when I was in admission and got discharged I would go back home and find another child sick. I would go back to the hospital and again be admitted. I was worried all the time and even feared I would lose my job due to the frequent absentia. We used to spend a lot of money on treatment, transport and food during the process. Sometimes my children would get sick during the night and we had to seek treatment at the private clinic because we would not have available transport to take us to the hospital. With all that we were going though, we wanted a solution that would save us from the situation but we didn’t have any. Malaria could not even spare me when I was pregnant.*

*In the process of all that one day the health workers advised us to use nets if we were to reduce on the malaria we were getting. Indeed it didn’t take my husband long to buy nets for the children and ourselves. We immediately started using the nets and we realised malaria was reducing in the home. My daughters who used to get convulsions started getting milder malaria. The next two pregnancies I never suffered from malaria and these two children never suffered from malaria like the first four. They have never slept without nets ever since they were born. This brought me joy and we had settled minds. Ever since that, we continue sleeping in nets. One thing I first pack for my children when they are going to school is a mosquito net. My children are now healthier and happy. My children now attend school regularly and they are performing well. With nets our future is bright, mostly my children’s future.”*

Box 3: Village Health Team (VHT) member, Bukomero A village, Kiboga (story no. 54).

*“Malaria has reduced in our village because I no longer see parents bringing their children to me to treat malaria. They now come for other diseases like cough unlike before when I would have to treat at least one child every day suffering from malaria.*

*The most significant change is that malaria has reduced especially among children and pregnant women. Before the introduction of nets, children would get severe malaria characterised by convulsions and even when they would bring them to us, we would fail to treat them and instead refer them to the health centre. This would lead to expenditure in terms of buying drugs and also transporting the patient to the health centre and as a result of this, a lot of time that should have been put to productive work like farming would be instead be diverted to caring for the patient hence contributing to poverty. For pregnant women, malaria was the lead cause of miscarriages until women started using nets. Even other insects like cockroaches have reduced.*

*However, with the introduction of nets, malaria has tremendously reduced and with the presence of VHTs in the village, people have been guided on how to use and maintain mosquito nets and this has improved on retention and usage. I think it is because of this reason that we have not yet hard any cases of nets burning in the house or misuse of nets in the community. We VHTs are trying as much as possible to ensure that people understand the importance of using nets by educating them on how to handle and manage nets in their homes.”*
